# Targeting Anabolic Impairment in Response to Resistance Exercise in Older Adults with Mobility Impairments: Potential Mechanisms and Rehabilitation Approaches

**DOI:** 10.1155/2012/486930

**Published:** 2012-09-11

**Authors:** Micah J. Drummond, Robin L. Marcus, Paul C. LaStayo

**Affiliations:** Department of Physical Therapy, University of Utah, 520 Wakara Way, Salt Lake City, UT 84108, USA

## Abstract

Muscle atrophy is associated with healthy aging (i.e., sarcopenia) and may be compounded by comorbidities, injury, surgery, illness, and physical inactivity. While a bout of resistance exercise increases protein synthesis rates in healthy young skeletal muscle, the effectiveness of resistance exercise to mount a protein synthetic response is less pronounced in older adults. Improving anabolic sensitivity to resistance exercise, thereby enhancing physical function, is most critical in needy older adults with clinical conditions that render them “low responders”. In this paper, we discuss potential mechanisms contributing to anabolic impairment to resistance exercise and highlight the need to improve anabolic responsiveness in low responders. This is followed with evidence suggesting that the recovery period of resistance exercise provides an opportunity to amplify the exercise-induced anabolic response using protein/essential amino acid ingestion. This anabolic strategy, if repeated chronically, may improve lean muscle gains, decrease time to recovery of function during periods of rehabilitation, and overall, maintain/improve physical independence and reduce mortality rates in older adults.

## 1. Aging, Muscle Atrophy, and the Clinical Significance

Preserving physical function, mobility, and ultimately the physical wellbeing of older adults is a high priority given the rapid rise in the number of older adults (>65 y) expected in the ensuing decades [1]. A common feature of aging that contributes in part to physical dysfunction is a slow but significant decline in muscle mass, especially high-quality force-contracting muscle fibers, beginning as early as the 4th or 5th decade of life [2, 3]. 

Older adults are particularly susceptible to accelerated muscle loss, following an acute catabolic event, such as physical inactivity [4–6] or surgery [7] and is followed by a less than optimal muscle recovery [5, 8]. For that matter, interventions geared toward older adults with various clinical pathologies that are on a progressive downward decline toward frailty (e.g., hip fracture, postoperative, pneumonia) are needed, especially when muscle mass reserves and quality are low, mobility impairments are high, and physical independence is dwindling. Although pharmacologic approaches are being investigated as alternative methods to increase or attenuate declines in muscle mass [9] few, if any, countermeasures are superior to resistance exercise. 

## 2. Exercise Is Good, But Is It Optimal?

Resistance exercise is considered an efficacious, cost effective treatment to promote muscle size, quality, and strength in older men and women. The American College of Sports Medicine [10] and two recent meta-analyses [11, 12] provide evidence that older adults increase lean mass and strength from high volume, moderate-high intensity resistance exercise programs over at least 12 weeks duration. Even in the oldest of old (80 and older) [13, 14] and those with limited mobility [15–17] high-intensity resistance exercise can induce muscle growth.

**Figure 1:**
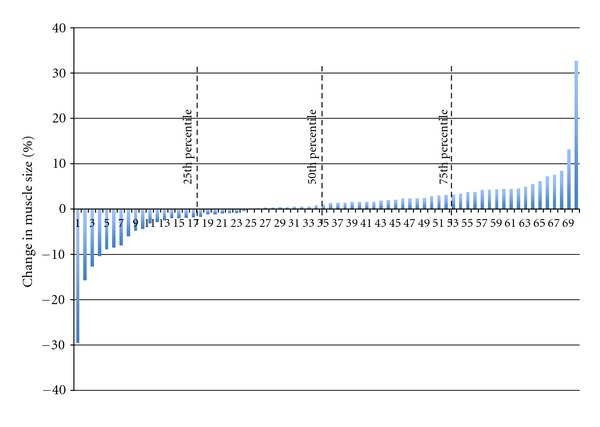
Individual percentage and quartiles of muscle change following 12 weeks of a multicomponent resistance exercise training program in mobility impaired older adults (*N* = 70, older (age 73 ± 6 y)).

Although older skeletal muscle is clearly capable of improving with resistance training, the capacity to respond is reduced thereby producing smaller muscle and strength gains when compared to younger peers [18–20]. For example, work from the Trappe Laboratory [21, 22] reported, in older men and women > 80 years old, an impaired ability to stimulate muscle fiber hypertrophy compared to younger adults following a resistance exercise training program. This notion has been substantiated by a thorough examination of 49 chronic resistance exercise training studies showing a negative association between age and lean body mass [12]. Certainly no one would argue that resistance exercise is beneficial and adaptations, albeit smaller and possibly more variable[23], are important to maintain physical independence for healthy older adults. However, the concern is for the most needy older adult groups who demonstrate limited response to a resistance exercise stimulus. These “limited or low-responders” [24] often includes the frailest and oldest of old. For instance, in a large cohort of mobility-impaired older adults (>70 y; *n* = 70) we noted significant variability in muscle size changes after 12 weeks of progressive resistance exercise (Figure 1). Approximately, 75% of these individuals had little to no muscle mass increase (<3%); profoundly different from what is typical in young and even in healthy older participants [25]. Clearly, for many older individuals the capacity to grow muscle and demonstrate positive muscle responses after resistance exercise is blunted.

The focus of this paper is to discuss how muscle mass is regulated in healthy, young individuals and highlight the blunted anabolic response to resistance exercise observed in older adults. We will also discuss potential mechanisms for this diminished response and provide evidence that the anabolic response to resistance exercise in older adults, and particularly mobility impaired older adults, can be enhanced with a cost effective, noninvasive, nonpharmaceutical approach: ingestion of protein/essential amino acids. 

## 3. Muscle Mass Regulation: A Basic Primer

**Figure 2:**
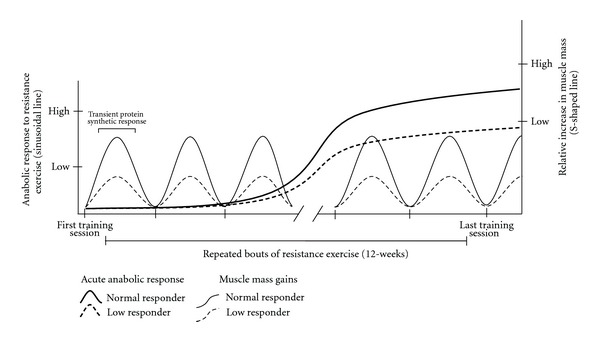
Theoretical model of anabolic response to resistance exercise in “normal” and “low responders”. Each sinusoidal line represents anabolic response (i.e., protein synthesis) to a bout of resistance exercise in normal (solid) and low (dashed) responders. S-shaped line represents muscle mass gains following 12 weeks of repeated bouts of resistance exercise in normal (solid) and low (dashed) responders.

Maintenance of skeletal muscle is fine-tuned by an intricate balance of proteins that are synthesized and broken down. A chronic daily imbalance in the ratio of protein synthesis/breakdown rate will to lead to gains (ratio > 1; positive protein balance) or losses (ratio < 1; negative protein balance) in muscle mass. Of this two-part equation, muscle protein synthesis is the most dynamic throughout the course of the day as contractile load and amino acids (primarily essential amino acids (EAA)) transiently increase protein synthesis in human muscle [26, 27]. However, in cases of catabolic diseases and conditions, protein breakdown may play a larger role in controlling protein turnover [28–31]. Resistance exercise acutely stimulates muscle protein synthesis in healthy young human skeletal muscle [32, 33]; a response that has been shown to be elevated at least 48 hours after a single bout of exercise [34]. Although not directly proven, the current dogma is that the cumulative acute increases in muscle protein synthesis from repeated bouts of resistance exercise are partly responsible for observable changes in muscle size [35] (Figure 2). While acute stimulation of protein synthesis is not the only factor contributing to muscle hypertrophy (e.g., satellite cells), we believe it is important and is central to the discussion in this paper.

From a cellular perspective, stimulation of protein synthesis by contraction and EAA in human muscle is largely regulated by the mammalian target of rapamycin (mTOR) [36, 37]. Activation of mTOR phosphorylates key proteins such as S6 kinase 1 (S6K1) and eukaryotic initiation factor 4E binding protein 1 important in the enhancement of mRNA translation initiation and elongation [38, 39]. Resistance exercise stimulates phosphorylation of several mTOR cell signaling intermediates within minutes of contraction [40]. Importantly, acute phosphorylation of proteins associated with mRNA translation has been causally linked to the skeletal muscle hypertrophy response. This can be found in rodent [41] and human [42–44] studies demonstrating a possible relationship between the magnitude of acute increases in translation initiation biomarkers (i.e., S6K1 phosphorylation, eukaryotic initiation factor 2B*ε*) following a single bout of resistance exercise to that of muscle size following a resistance exercise training program. 

## 4. Potential Mechanisms of Age-Induced ****Anabolic Insensitivity to Exercise

It is unlikely that age-related muscle loss in healthy humans is a result of diminished resting rates of protein synthesis and/or increased protein breakdown [45] as muscle loss would be much more rapid and apparent. However, we do not want to dismiss the point that a catabolic event(s) may target resting protein turnover rates. It is more likely that healthy older adults and those in diseased or catabolic conditions are less responsive to the anabolic stimulation of amino acids [46–50] and resistance exercise. To keep this paper focused, we would like to discuss the lines of evidence indicating that older adult skeletal muscle has a diminished capacity to respond anabolically to a single bout of resistance exercise—a term described as “anabolic impairment”. Reports indicate a decreased ability to stimulate an increase in muscle protein synthesis following an unaccustomed bout of high-intensity resistance exercise in healthy older adults [43, 51, 52]. It is hypothesized that the blunted increase in protein synthesis following acute muscle loading repeated chronically may be partly responsible for the smaller gains in lean tissue following resistance exercise training in older adults. However, this theory needs to be supported with additional studies in older adults capturing muscle protein stimulation responses following subsequent acute bouts of resistance exercise. 

It is not completely understood why acute synthesis rates are less robust following a single session of resistance exercise in older versus younger adults, but it is not surprising that mTOR and its downstream targets have been considered likely suspects. Kumar et al. [51] was the first to examine this relationship. In this study, it was noted at a range of exercise intensities (60–90% one repetition maximum) that, in addition to impaired muscle protein synthesis rates, phosphorylation of S6K1 and 4EBP1 failed to increase 1–4 h following a bout of resistance exercise in older adults. Recently, our work has noted that a blunted increase in protein synthesis and mTOR signaling persisted at least 24 h after resistance exercise, involved mitogen-activated protein kinase signaling, and occurred in both healthy older men and women [52]. In another study, Greig et al. provided clues that a failure to downregulate REDD1 mRNA (codes for a potent protein inhibitor of mTOR) following an acute bout of maximal isometric resistance exercise may be partly responsible for the smaller muscle mass increases in older (~80 y; 2.5%) versus younger (~26 y; 6.2%) women after 12 wk of maximal isometric exercise training [19]. This latter study implies that negative mTOR regulators, such as REDD1 and others, should be investigated further in efforts to explain or correct the impaired stimulation of mTOR and protein synthesis in older skeletal muscle. The thought of monitoring acute changes in REDD1 as a surrogate marker of chronic hypertrophy responses is intriguing. 

While there is evidence that oxidative stress may negatively impact muscle protein synthesis following immobilization (at least in old rodent muscle) [53], several studies strongly point to inflammation as a potential contributor to blunted muscle protein synthesis rates. This has been observed at rest and in response to amino acids in older humans and animals and as a result of immobilization [8] and catabolic diseases [47–49]. For instance, Rieu and colleagues provided a daily dose of ibuprofen for 5 months in old rats with low-grade inflammation. After the treatment, feeding-induced stimulation of muscle protein synthesis was enhanced whereas feeding had no effect on muscle protein synthesis rates in old rats without ibuprofen treatment [54]. Mechanistically, inflammation may influence protein synthesis through inhibition of translation initiation by proinflammatory mediators, such as tumor necrosis factor (TNF*α*) [55]. This is very interesting and potentially impactful in older adults with clinical pathologies since chronic low-grade systemic and local inflammation is associated with aging [56, 57], heightened with disease and inactivity [57, 58], and is related to muscle and strength losses [59, 60]. However, no study to date has identified if chronically elevated levels of inflammation at rest in humans adversely impact the muscle anabolic sensitivity to acute resistance exercise and whether this occurs through the mTOR pathway. At best, Greiwe and workers [61] indicated that postabsorptive muscle protein synthesis rates after resistance exercise training were inversely related to TNF*α* muscle content in frail older adults. While in another study, Trappe and colleagues [62] identified that a chronic combination of resistance exercise and anti-inflammatory drugs improved muscle and strength gains in healthy older adults compared to those who exercise-trained independent of pharmacological intervention. Certainly inflammation is both a normal and necessary response for muscle remodeling [63] and the hypertrophy process [64]. The question remains, however, whether there is an unhealthy level of inflammation that may adversely impact the capacity to respond to resistance exercise [65] like what is observed with the anabolic responsiveness to amino acids [47–49, 54]. To this point, identifying strategies to improve anabolic sensitivity to resistance exercise is of upmost importance in older adults who are healing from injury or surgery and who have low-muscle reserve (frail, hip fracture) as these are important target populations in which muscle growth may be further compromised (Figure 2).

## 5. Amplifying the Anabolic Response ****to Exercise

Muscle protein synthesis can be enhanced by acute ingestion of EAA or high-quality protein (e.g., lean beef, whey) even when uncoupled from resistance exercise [26, 66, 67]. Of the EAAs, leucine has been highlighted as a potent anabolic nutrient that is not only used as a substrate for synthesis of new proteins but also as an anabolic trigger for mTOR-related signaling events [68]. However, the anabolic response to protein/EAA ingestion is short lived (~3–5 h) and does little benefit to the hypertrophy process in healthy skeletal muscle independent of chronic contractile loading.

When resistance exercise is performed independently of nutrient intake, protein breakdown exceeds synthesis rates and protein balance becomes negative [33, 34]. However, when nutrients in the form of EAA or protein are provided in close proximity to a bout of resistance exercise muscle protein balance is positive [69, 70] and augments the acute muscle protein synthesis response [71–73] with corresponding increases in mTOR signaling [74, 75]. Furthermore, resistance exercise training combined with protein supplementation in young individuals have demonstrated increases in muscle mass more so than resistance exercise alone [76–78].

More importantly the amplified anabolic response of a single bout of resistance exercise combined with EAA or protein intake are also recognized in older adults [79–83]. For example, a recent study by Yang et al. [84] showed that healthy older adults demonstrate a dose-dependent stimulation of protein synthesis when 20 g–40 g of whey protein were taken immediately following exercise. Although this study did not directly conduct a side-by-side comparison to young individuals, other investigations have verified that the acute protein synthetic response in older adults is capable of being similar to the young [79, 82, 83]. These data support the notion that an impaired anabolic response to an acute bout of resistance exercise in older adults can be rescued to youthful levels when followed with an adequate dose of EAA or high-quality protein.

There are far fewer chronic resistance exercise studies with protein supplementation in older adults compared to younger individuals. While some studies have shown increases in lean mass in older adults following 12 weeks of resistance exercise with post-exercise protein ingestion they have lacked an adequate control group (exercise with no supplementation) to come to an adequate conclusion [85, 86]. In studies that have utilized a control, the superiority of supplementing resistance exercise training with a protein or EAA source is not evident in healthy older adults [87–89]. Most recently, Verdijk et al. [90] provided a 10 g protein supplement (casein hydrolysate) before and immediately after a bout of resistance exercise in healthy, nonsarcopenic, older adults over a 12-week resistance training program. Although there were significant muscle size and strength gains, these improvements were no different from the control group devoid of the supplement. As these authors explained, the benefits of providing a postexercise protein supplement may not be necessary in older adults who meet/exceed their dietary protein requirements. In another study, Fiatarone and colleagues examined the benefits of combining resistance exercise with a liquid nutrient supplement (360 kcal; 60% CHO, 23% fat, 17% protein) in institutionalized older adults (age: ~87 y) [91]. Again, resistance exercise training was beneficial to improve strength and muscle size but the addition of a nutritional supplement did not further improve outcomes. However the timing of the nutrient supplement was not provided in close proximity of their training session. Perhaps, in view of the work of Yang et al. [84], it is plausible that healthy older adults need a larger high quality protein supplement (~40 g; ~20 g EAA) that is provided in close proximity to each exercise bout to maximize muscle hypertrophy and strength benefits following resistance exercise training. 

It is possible that using a potent anabolic intervention (resistance exercise + protein/EAA supplement) may be beneficial in those attempting to regain lean tissue during physical rehabilitation, that is, injury/surgery or illness, but not necessarily in circumstances of slow atrophy from aging. A study by Holm and colleagues [92] found that individuals who experienced a recent ACL injury had improvements in muscle size and strength after 12 weeks of resistance exercise training when a 10 g protein (milk + soy) and carbohydrate supplement were provided before and after each bout of resistance exercise [92]. Typical of this patient population, these individuals were classified as “young” (~25 y) and these results cannot be generalized to an older population. Certainly more studies using resistance exercise combined with protein/EAA supplementation are needed in older adults with compromised muscle function and mobility that are in a bad condition following an acute catabolic event (i.e., injury, illness, infection). Perhaps an improved anabolic sensitivity to repeated bouts of resistance exercise during physical rehabilitation may result in more individuals fully recovering from a catabolic event. In implementing this anabolic strategy, investigators should keep in mind the quality of protein [93], the protein dose [84, 94], the timing of the supplement [85], and the length of the resistance exercise + protein supplementation trial (≥12 weeks).

## 6. Summary & Conclusion

In summary, resistance exercise training is a suitable means to increase muscle size, strength, and mobility in older persons. Despite these improvements, the gains from resistance exercise are variable and relatively smaller than those seen in younger adults. That is, many older adults are low responders and have a reduced anabolic capacity to resistance exercise and this blunting is perhaps even more apparent in older adults with clinical pathologies. A plausible mechanism underlying this blunted resistance exercise response may be an impaired stimulation of muscle protein synthesis mediated in part by cell-signaling events that impact mRNA translation (e.g., mTOR). Providing a protein/EAA source during the early after exercise period can further enhance the anabolic sensitivity (i.e., protein synthesis, mTOR signaling) of resistance exercise in older adults. In healthy older adults with adequate dietary protein intake, there do not appear to be further benefits of complementing resistance exercise with a protein/EAA source. Although this may be the case in healthy older adults who do not have overt sarcopenia, who are physically independent and have adequate protein intake, the benefits of amplifying anabolic sensitivity in more vulnerable, mobility impaired older adults remains inconclusive and deserves further investigation.
